# Fecal microbiota of different reproductive stages of the central population of the lesser-long nosed bat, *Leptonycteris yerbabuenae*

**DOI:** 10.1371/journal.pone.0219982

**Published:** 2019-07-18

**Authors:** Osiris Gaona, Elizabeth Selene Gómez-Acata, Daniel Cerqueda-García, Carla Ximena Neri-Barrios, Luisa I. Falcón

**Affiliations:** 1 Posgrado en Ciencias Biológicas, Universidad Nacional Autónoma de México, Instituto de Ecología, UNAM, Mexico City, México; 2 Laboratorio de Ecología Bacteriana, Instituto de Ecología, Universidad Nacional Autónoma de México, UNAM, Parque Científico y Tecnológico de Yucatán, Mérida, Yucatán, México; 3 Consorcio de Investigación del Golfo de México (CIGOM), Centro de Investigación y de Estudios Avanzados del Instituto Politécnico Nacional, Unidad Mérida, Departamento de Recursos del Mar, Mérida, Yucatán, México; Kyoto University, JAPAN

## Abstract

In this study we analyzed the microbiota composition of fecal samples from the lesser-long nosed bat *Leptonycteris yerbabuenae* in different reproductive stages (juveniles and adult bats of both sexes as well as pregnant and lactating females). The V4 region of the 16s rRNA gene from 33 individuals was analyzed using alpha and beta diversity metrics. We found that microbiota diversity (expressed in Amplicon Sequence Variants) is higher in pregnant and lactating females. The microbiota of the juveniles and non-reproductive adults was dominated by Gammaproteobacteria and Firmicutes. Reproductive females had a much more diverse microbiota, with a significant increase in phyla such as Bacteroidetes and Alphaproteobacteria. There was no difference in fecal microbiota diversity between pregnant and lactating females and juveniles and non-reproductive adults. Results suggest that differences in microbiota diversity are related to reproduction. We infer that males maintain stable microbiota composition because they do not undergo the large physiological changes that females do during reproduction and maintain a more specialized diet throughout all life stages.

## Introduction

The community of microorganisms that reside in the vertebrate gut executes a variety of functions that impact host phenotype, nutrition, detoxification of xenobiotics, gut stimulation, immune development and behavior [1,2,3]. Thus, bacteria are directly involved in their hosts’ fitness. In particular, bacteria can improve energy assimilation from different food sources through the synthesis of vitamins necessary for physiological functions [4]. For instance, mammalian genomes do not encode most of the enzymes needed to degrade the structural polysaccharides present in plant material [5], leaving them dependent on symbiotic gut microorganisms that are capable of accessing different sources of energy. Herbivores and omnivores benefit from additional energy from microbial fermentation of carbohydrates in the gut [6]. This has led to major changes in digestive anatomy and physiology that allow efficient microbial fermentation to take place alongside the recovery of dietary energy by the host [6]. In some vertebrates, such as ruminants, gut microbes are so essential that hosts coevolved specialized organs to enhance gut microbial functionality [7].

Studying the microbiotas of wildlife is difficult, since there are many variables that are likely to impact microbiota that cannot be controlled. For example, diet has been demonstrated to be a main factor shaping the functionality and diversity of the gut microbiota, resulting in convergent microbial communities among hosts with similar feeding habits [8,9]. There is evidence that diet shapes the relative abundance of dominant phyla, and populations of specific bacterial groups are influenced by the composition of macronutrients consumed; in addition, food itself can serve as a reservoir for new microbial introductions [10,11]. Empirical studies show that microbiota respond rapidly to changes in host diet [12,13].

Animals’ dietary needs change depending on their life-history stage. In the genus *Nasonia*, the microbiota differs among the three developmental stages present in this genus, particularly between the pupal and adult stages [14]. As mentioned above, changes in the microbiota can be due to a variety of factors that may change among life stages, including environmental conditions, diet, weight, and hormones [15].

During pregnancy, there are various hormonal, immune, and metabolic changes that are associated with increases in the bacterial load in several organs, including the vagina, oral cavity, and intestine [15]. In the case of *L*. *yerbabuenae*, there is a significant change in females’ feeding habits during reproduction, with pregnant and lactating females consuming an increased diversity of plants [16]. These changes may be temporary, related to the preferences and nutritional requirements of the individual during different reproductive stages [16]. Reproductive females of *L*. *yerbabuenae* and other females of nectarivorous bats (*Glossophaga soricina*) have been reported as active feeders on flowers in the driest season of the Mexican central highlands when flowers are the only food resource [17].

Microbes that reside in the gastrointestinal tract respond dynamically as a community to those changes over an individual’s lifespan [10]. In small mammals, the direct costs of pregnancy and lactation include increased energy, protein and calcium demands. Organ re-modelling is necessary to achieve the high demands of lactation and involves growth of the alimentary tract and associated organs such as the liver and pancreas [18]. The pre-natal period is shaped by immunological and inflammatory changes that modify the functionality of the gut and bacterial composition in females as much as pregnancy [19,20], while in non-pregnant healthy females microbiota composition has been reported to be relatively stable [21]. Another factor affecting the functionality of the gut during the pregnancy and pre-natal stages are the hormones estrogen and progesterone [19]. Geography and behavior can also shape microbial composition. For example, the microbiota of an isolated human population of hunter-gatherers in Hadza, Tanzania presents a cyclical succession of bacterial species that correspond to the richness of functions associated with the season, and which differs from that of urbanized communities [22]. This population has limited access to plant-based foods and a carbohydrate-rich diet [23,24]. In amphibians, the biodiversity of the microbiota has been shown to vary within species depending on the geographic position along a river [25]. Some authors describe the host as a topographic map, since physical and chemical variables change along different parts of the host’s body, including pH, texture, salinity, and sebaceous content; these variables are determined by factors that are intrinsic to the host (e.g. genotype, age, sex), as well as factors that are extrinsic but depend on the individual (e.g. in humans, occupation, lifestyle, geographic location, and use of antibiotics or cosmetics) [26, 27]. The host microbiota can also be shaped by individual behavior [28]; social animals acquire much of their resident bacterial population directly through social grooming of their group members or indirectly from the environment [28].

*Leptonycteris yerbabuenae* is a migratory nectar specialist bat that is a pollinator of columnar cacti and Agave in North America [29]. Unlike other bat species such as *Phyllostomus hastatus* [30] or *Artibeus jamaicensis* [31] which carry out all of their activities and spend their whole life cycles in a single roost, *L*. *yerbabuenae* has a more complex life cycle in that it uses geographically separate roosts for copulation, giving birth, and rearing young, with roosts occupied by adult males and females [32–37]. This species is migratory, and has different roosts where they will complete their life cycles, which are limited by food availability [17].

**Fig 1:**
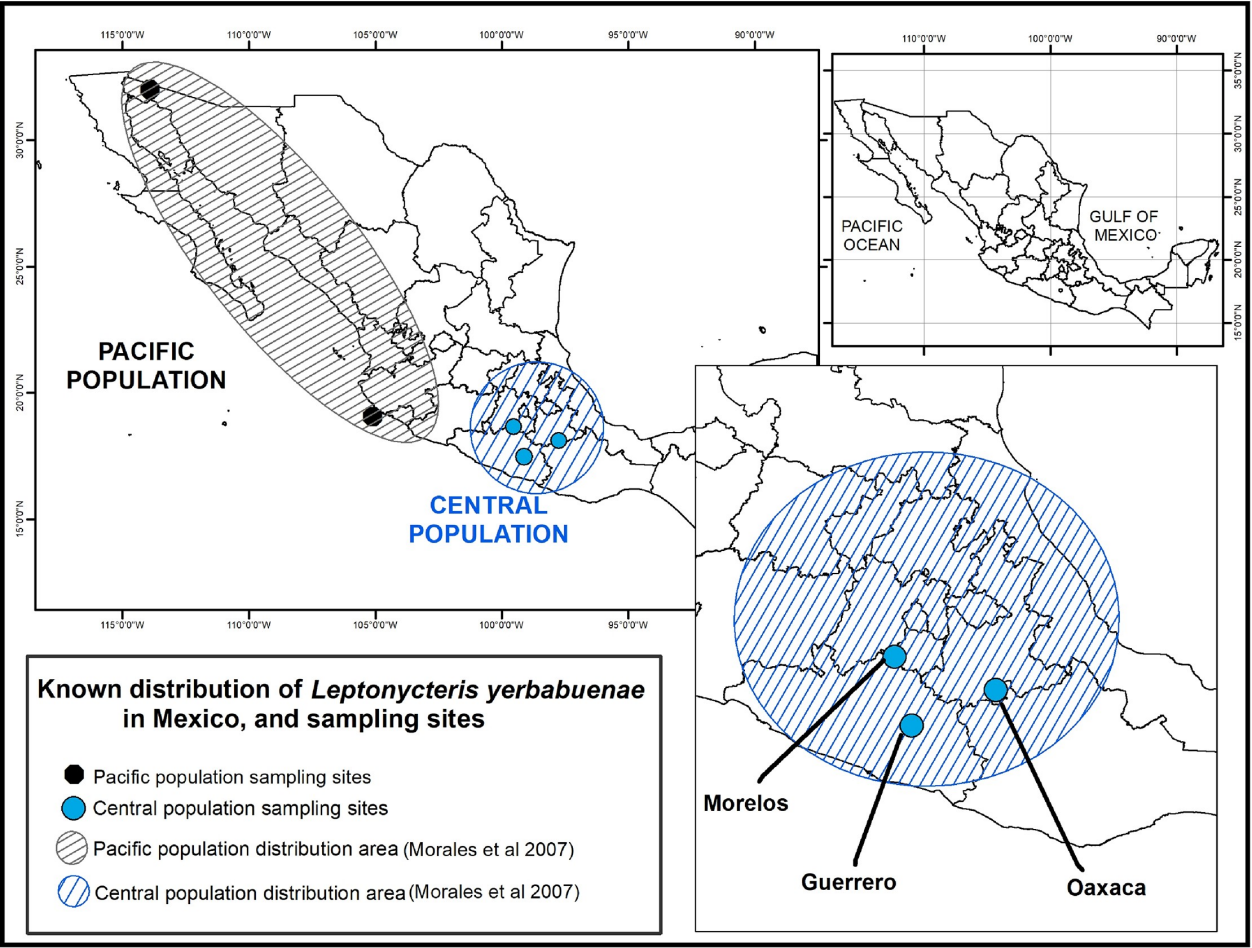
Distribution of the two differentiated *L*. *yerbabuenae* populations in Mexico. The Pacific population is found in the states of Baja California, Sonora and Jalisco. The Central population (from which our samples were collected) is found in Morelos, Guerrero and Oaxaca.

*L*. *yerbabuenae* has two differentiated populations in Mexico: one along the Pacific coast including Baja California, Sonora and Jalisco, and a central population that occurs in south-central Mexico, in the states of Oaxaca, Morelos, and Guerrero [38–41]. This central population has local migration patterns, but is considered a resident population since there is constant food availability due to the large diversity of cacti in the region [38,39] (Fig 1). In this study we collected fecal samples from the central population of the lesser-long nosed bat to explore the microbiota composition in different reproductive stages (juvenile and adult males and females, and pregnant and lactating females), in order to describe how the microbiota differs among different reproductive stages over the complex life history of this species. Our hypothesis is that pregnant females will have the highest microbiota diversity due to the dietary and physiological changes that occur during pregnancy.

## Methodology

### Study site

Bat fecal microbiota samples were collected at three caves previously known to host specific reproductive stages of the lesser long-nosed bat from the Central population, between January and November, 2015 (as reproduction progressed in *L*. *yerbabuenae* reproductive stages). Reproductive adult males were sampled in San Juan Noxchitlan, Oaxaca (97^o^ 40’ N and 18^o^ 03’ W), a colony of 100,000 resident bats [42] in June, 2015. Pregnant and lactating females were captured in Juxtlahuaca Cave, Guerrero (17^o^ 23” 3´ N and 99^o^ 16” 1´W) in November, 2015, and non-reproductive adult females were sampled in Salitre Cave, Morelos (18^o^ 45” 0.05´N and 99^o^ 11” 23.17´) in April, 2015 (Table 1). As mentioned above, because caves are segregated by life history stage, it is not possible to find all of the different life stages in a single cave, and roosts can change from year to year.

**Table 1:** Sampling data from different reproductive stages of L. yerbabuenae.

Reproductive stage	Feeding	Study Site	Sex	Number of bats
Lactating	Nectar	Guerrero	Female	6
Pregnant	Nectar	Guerrero	Female	6
Adult	Nectar	Morelos	Female	6
Adult	Nectar	Oaxaca	Male	11
Juvenile	Nectar	Oaxaca	Male	4

The colony in “San Juan Nochixtlan” cave where adult males were sampled, has a mean annual rainfall of ca. 400 mm with an average temperature of 21º C in a semi-arid region [43]. The region has a high number of columnar cacti species, containing 19 of the 45 reported for south-central Mexico [29]. The landscape surrounding the “Juxtlahuaca” cave is characterized by deciduous forest vegetation and seasonal maize cultivation [44]. The characteristic climate region is dry sub-humid warm weather with rainy season in summer, with mean temperatures between 20 and 29° C [44,45]. Pregnant and lactating females were sampled here.

The “El Salitre” cave, where non-reproductive adult females were sampled, is located at an altitude of 1140 masl, in a warm sub-humid climate region with an annual total of 800–1000 mm of rain concentrated in the summer, dry winters and a mean temperature of 22°C [44,46] in a deciduous forest environment, with fragments of secondary vegetation and sugarcane and maize fields [44,46,47].

### Bat fecal microbiota sampling

Bats were captured with 12m-long mist nets (Avinet, Dryden, New York, USA) at the entrance of the caves using Kunz’s technique [48], between 18:30 and 7:00 h. Each captured bat was individually placed into a clean sterile plastic bag, leaving a gap to ensure ventilation and avoid suffocation. Individuals defecated in a matter of seconds, and no chemical immobilizers, analgesics or sedatives, were needed. The fecal samples were collected using sterile 1.5 ml Eppendorf tubes and frozen in liquid nitrogen until they arrived at the laboratory, where they were stored at -80º C until processing. The bats were taken out of the bag to take standard body measurements and released *in-situ*. Total samples processed per reproductive stage are shown in Table 1.

Standard measurements of each individual included forearm length (measured using a manual caliper with a precision of 0.01 mm) and body mass (measured with a 100 g spring balance). Individuals’ age category (juvenile or adult) was estimated based on the ossification of wing bones (metacarpals and phalanges) [49]. The condition of testes (scrotal or inguinal) was recorded in males to determine whether they were reproductively active. Pregnancy and lactation was confirmed in females by palpation of the belly and mammary glands, respectively.

### Ethics statement

Samples were taken from wild bats that were released in the same area as capture immediately after fecal samples a body measurements were taken, causing no apparent harm to any of the individuals captured. *Leptonycteris yerbabuenae* is not under federal protection by Mexican law (NOM-059-SEMARNAT-2010). Scientific collection activities were carried out under a scientific collection permit number granted by the Mexican Secretary of the Environment and Natural Resources (SEMARNAT), number FAUT-0231, SGPA/DGVS/05780/15. SEMARNAT approved and authorized the tissue sampling methods under this collection permit. Laboratory activities were carried in the Ecology Institute of the Universidad Nacional Autónoma de Mexico (UNAM); no specific permit was needed because only tissue and skin samples were used (no *in-vivo* studies were included). All Biosecurity standard requirements from the Ecology Institute were satisfied.

### Extraction of DNA from feces

Metagenomic DNA was extracted from the fecal samples using the DNeasy Blood & Tissue kit (Qiagen, Valencia, CA) according to the manufacturer’s instructions. Briefly, feces collected into 1.5 ml sterile tubes were diluted with 180 μl of ATL extraction buffer with 20μl of proteinase K (10 mg ml^-1^), were mixed thoroughly by vortexing and incubated at 56°C at 1500 rpm for 50 min. 200μl of AL Buffer with 200 μl ethanol (96–100%) were added and mixed thoroughly by vortexing. The mixture was transferred into the DNeasy Mini spin column, washed with Buffer AW1 and then with AW2. The DNA was eluted with 200 μl of Buffer AE and precipitated with absolute ethanol, 0.1 volume 3 M sodium acetate and 2 μL glycoblue. DNA was resuspended in 30 μL of molecular grade water and stored at -20°C until PCR amplification.

### 16S rRNA gene amplification and sequencing

DNA samples were PCR amplified using the hypervariable region V4 of the 16S rRNA gene with universal bacteria/archaeal primers 515F/806R following the procedures reported by Caporaso et al. [50] and Carrillo et al. [51]. PCR reactions (25 μL) contained 2–6 ng of total DNA, 2.5 μL Takara ExTaq PCR buffer 10X, 2 μL Takara dNTP mix (2.5 mM), 0.7 μL bovine serum albumin (BSA, 20 mg ml^-1^), 1 μL primers (10 μM), 0.125 μL Takara Ex Taq DNA Polymerase (5 U μl^-1^) (TaKaRa, Shiga, Japan) and nuclease-free water. Samples were amplified in triplicate using a PCR protocol including an initial denaturation step at 95°C (3 min), followed by 35 cycles of 95°C (30 s), 52°C (40 s) and 72°C (90 s), followed by a final extension (72°C, 12 min). Triplicates were then pooled and purified using the SPRI magnetic bead, AgencourtAMPure XP PCR purification system (Beckman Coulter, Brea, CA, USA). The purified 16S rRNA fragments (~20 ng per sample) were sequenced on an Illumina MiSeq platform (Yale Center for Genome Analysis, CT, USA), generating ~250 bp paired-end reads. The sequence data are available from the NCBI Bioproject number PRJNA508738 with accession numbers SRR8303327 to SRR8303359.

### Analysis of sequence data

**Fig 2:**
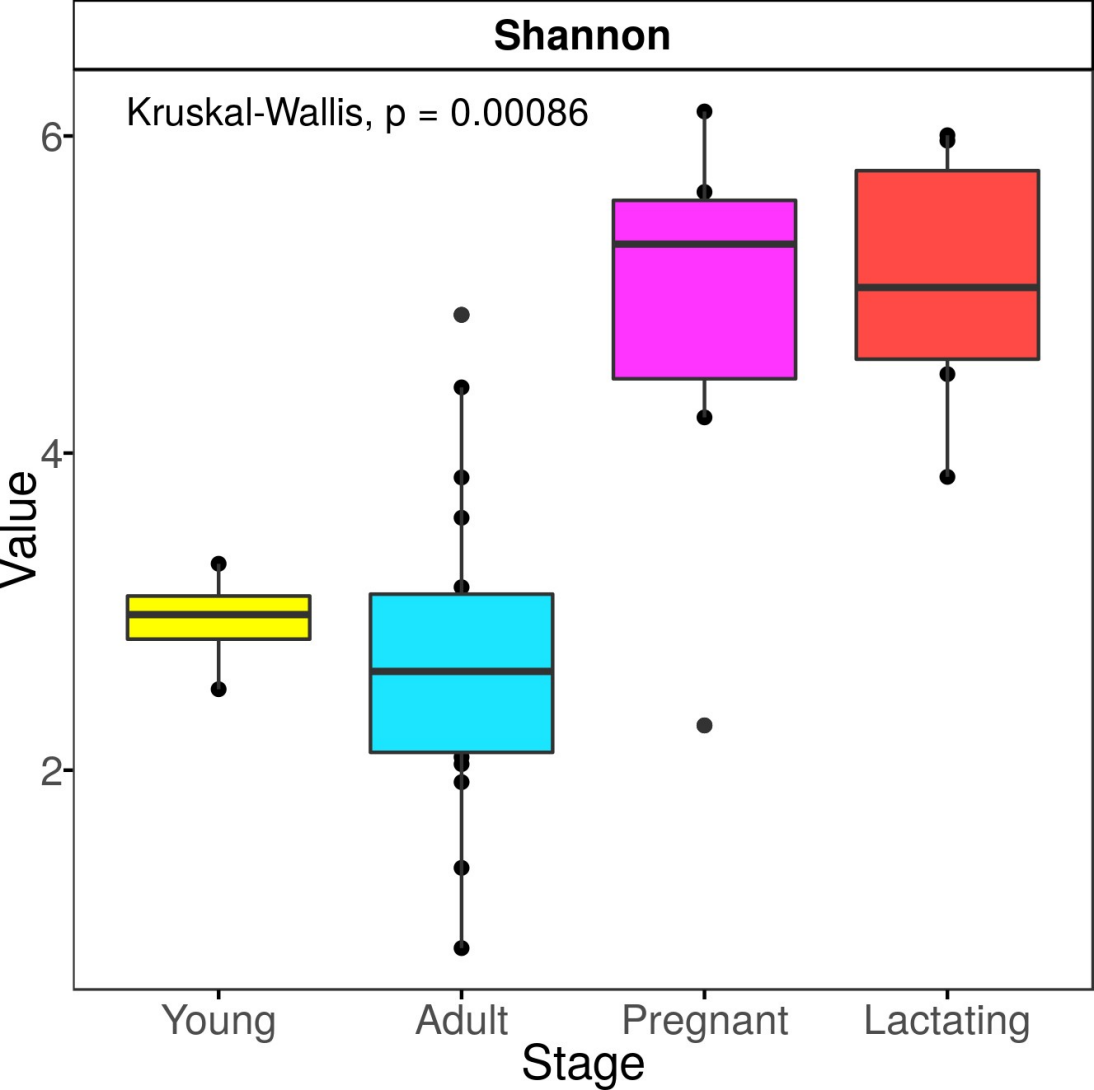
Alpha diversity index by reproductive stage.

Sequences were analyzed using the QIIME2 pipeline (v.2018.6) [50] (https://qiime2.org). Paired-end reads were demultiplexed with the Qiime plugin *demuxemp-paired*, then processed with the DADA2 plugin in the *denoise-paired* mode [52], trimmed at position 14 from the 5’ end, and truncated at position 200 from the 3’ end in both forward and reverse after manually verifying quality. Sequences were then denoised, and the amplicon sequence variants (ASVs) were resolved. Chimeric sequences were removed using the consensus method. Representative sequences of each ASV were taxonomically assigned using the QIIME plugin *feature-classifier classify-consensus-vsearch* (v 2.9.0) [53] searching in the SILVA database (release 132–99% OTUs, 515–806 region, L7 taxonomy) which was used to analyze the microbiota composition for each reproductive stage. The representative ASVs were aligned with the MAFFT algorithm [54]. After the subsequent masking of the positional conservation and gap filtering, a tree was built with the FastTree algorithm [55]. The feature table was rarified according to the same level of surveying effort of 12,569 reads per sample (S1 Fig). Mitochondrial and plastid sequences were filtered from all samples before rarefaction. The data was exported to the R environment (http://www.R-project.org/). Alpha diversity indices (Observed species, Shannon and Simpson index) were calculated with the phyloseq package [56] (Fig 2, S1 Table).

**Fig 3:**
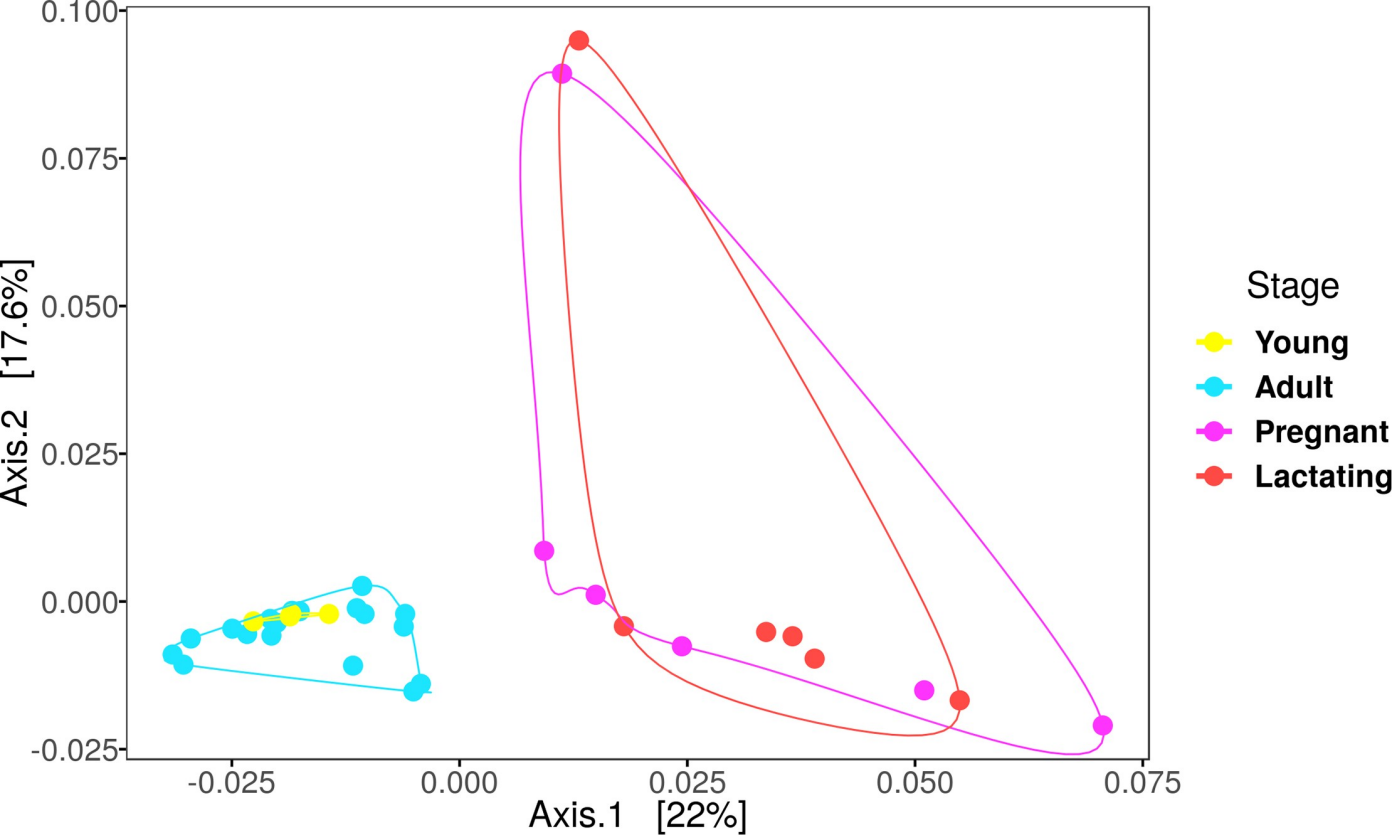
PCoA with the weighted unifrac distances by reproductive stage.

A Principal Coordinate Analysis was calculated with the weighted unifrac distance (Fig 3). To assess whether the differences between stages were statistically significant, a PERMANOVA was carried out using the vegan package [57] with the weighted unifrac distance matrix and 1000 permutations. A permutation test for homogeneity of variance (with betadisper and permutest functions) was carried out to assess the reliability of the beta diversity results.

A linear discriminant analysis (LDA) effect size (LEfSec) [58] was performed at the ASV level to find the microbial taxa whose abundances differed amongst reproductive stages, using an LDA cut-off of 2 and a Kruskal-Wallis alpha value of 0.01 (Fig 2).

## Results

### Microbiota composition during different reproductive stages

A total of 33 fecal samples were obtained and classified according to the sex, age and reproductive stage of the individual: Juvenile male, Adult male, Juvenile female, Adult (non-reproductive) female, Pregnant female, and Lactating female. Sample sizes per group are given in Table 1.

**Fig 4:**
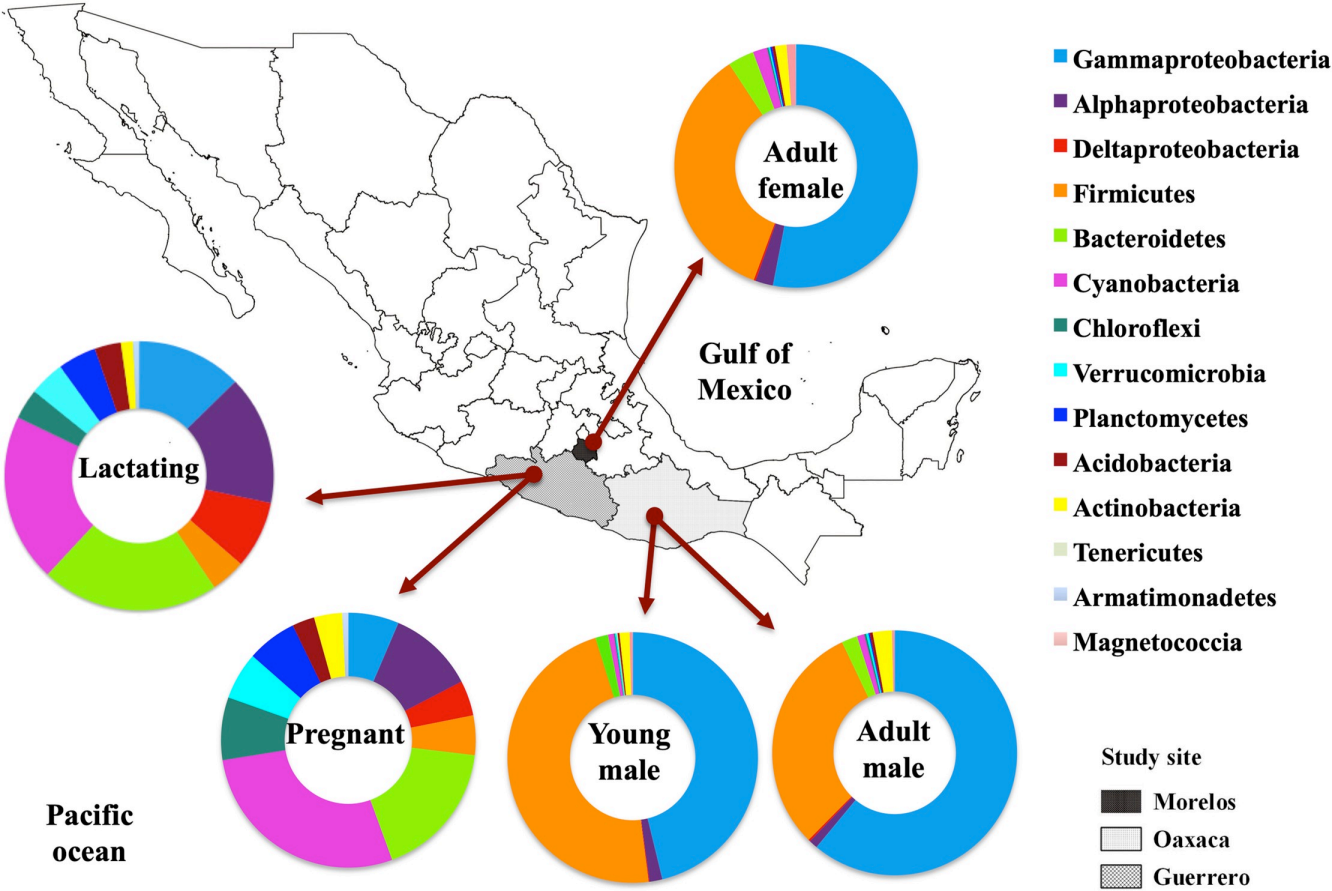
Distribution of bacterial phyla, class and order composition of fecal microbiota of *L*. *yerbabuenae* in different reproductive stages.

A total of 5,980,446 16S rRNA gene reads were obtained. After quality filtering, this was reduced to 4,120,896 reads. We found a total of 41 bacterial phyla in the bats’ fecal microbiota. The dominant phyla in the samples as a whole were Firmicutes, Proteobacteria (principally class Alphaproteobacteria, Gammaproteobacteria and Deltaproteobacteria), followed by Bacteroidetes, Chloroflexi, Planctomycetes, Verrumicrobia, Acidobacteria, Tenericutes and Cyanobacteria in reproductive (pregnant and lactating) females (Fig 4).

The most abundant phylum in lactating females was Proteobacteria (35%) followed by Bacteroidetes (21%) and Cyanobacteria (20%). Pregnant females’ gut microbiota was dominated by the phylum Cyanobacteria (27%) followed by Proteobacteria (21%) and Bacteroidetes (17%). The microbiota of non-reproductive adult females, adult males, and juveniles was dominated by the phylum Proteobacteria (54%, 62% and 48% respectively), followed by Firmicutes (34%, 30% and 47% respectively) (Fig 4).

**Fig 5:**
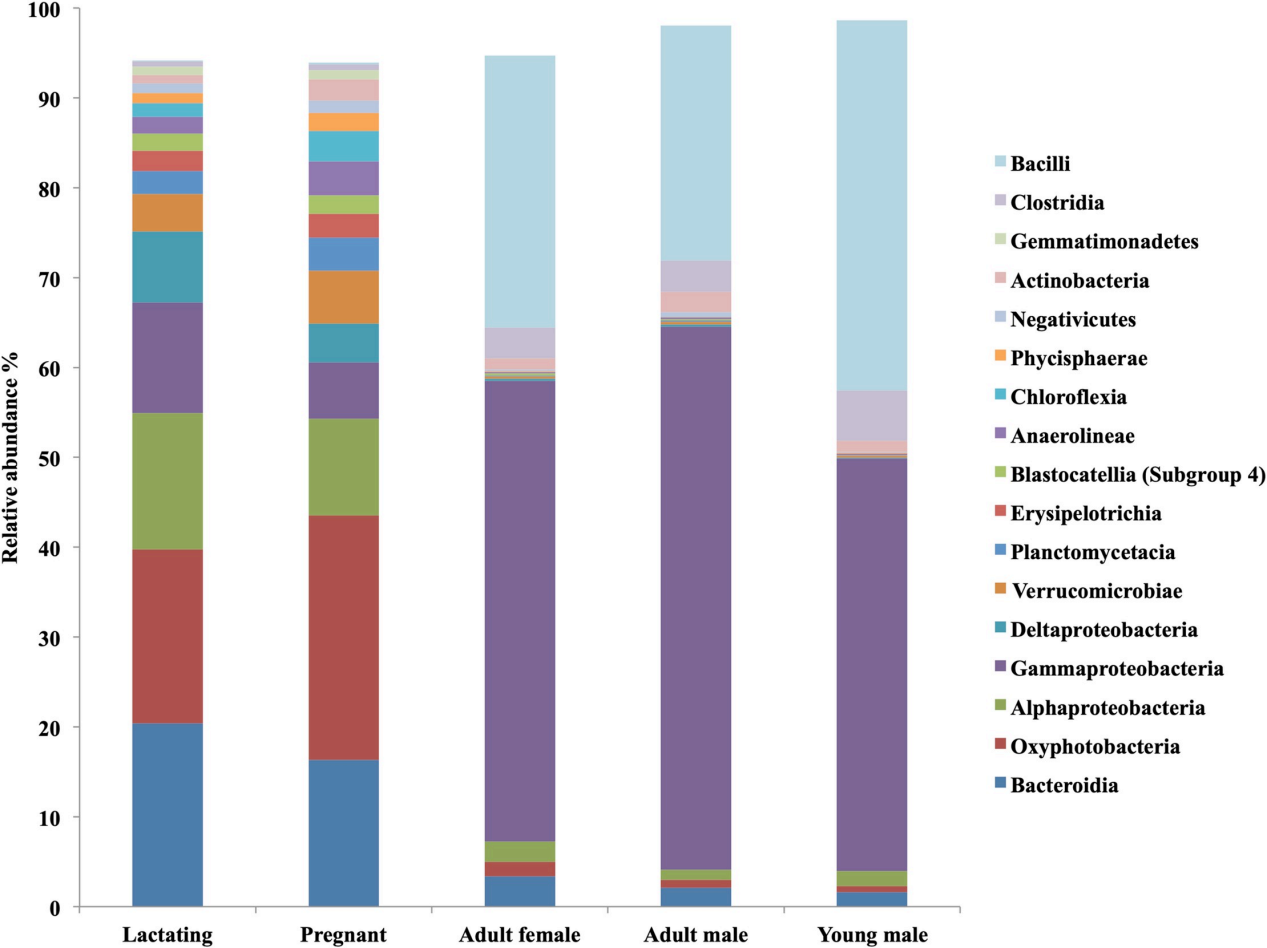
Distribution of bacterial Class composition of fecal microbiota of *L*. *yerbabuenae* in different reproductive stages.

**Fig 6:**
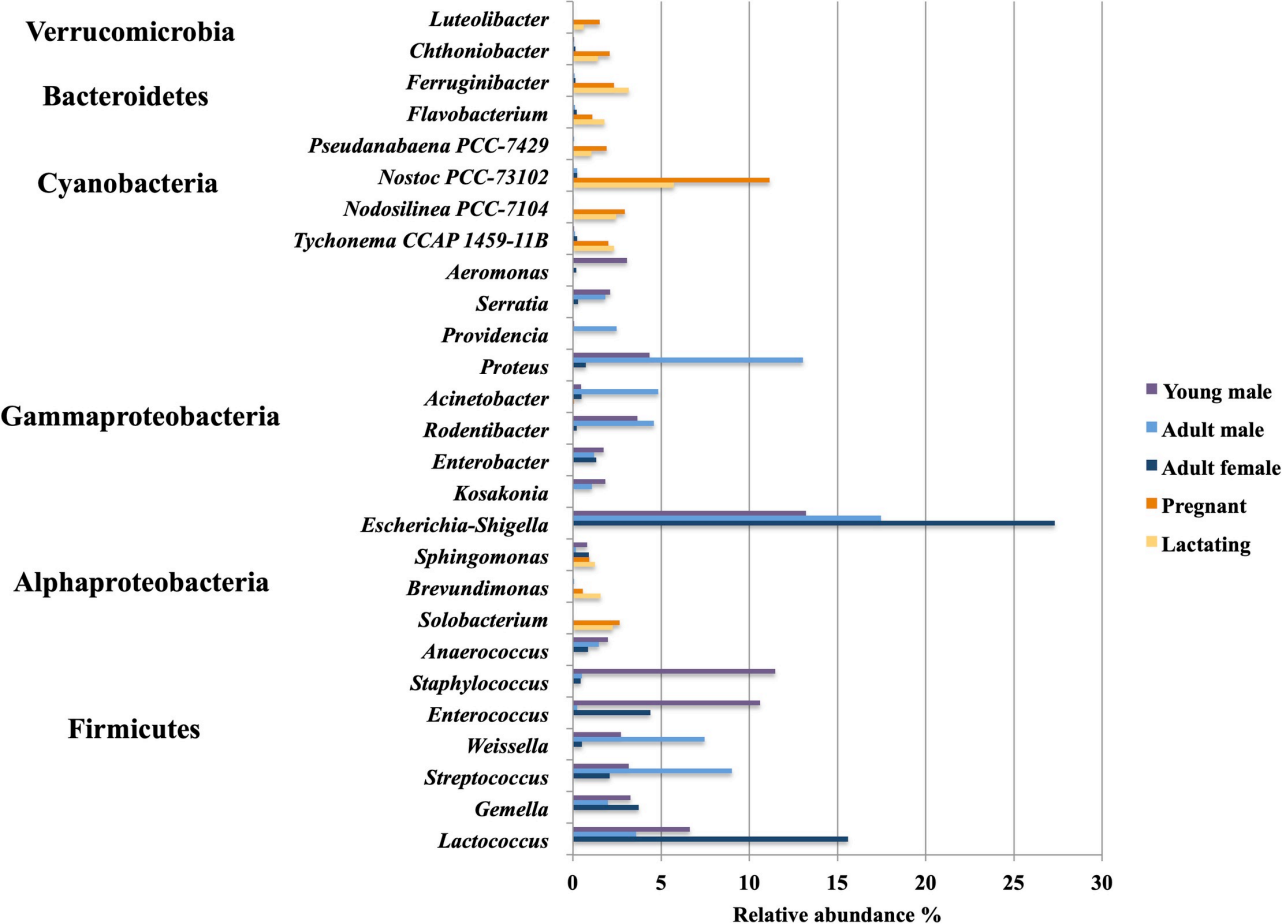
Distribution of bacterial Genus composition of fecal microbiota of *L*. *yerbabuenae* in different reproductive stages.

**Fig 7:**
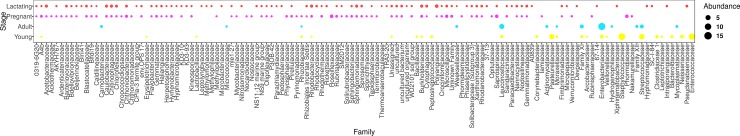
Linear discriminant analysis (LDA) effect size (LEfSec) showing similarities in the microbial communities of *L*. *yerbabuenae* at the family level.

Lactating females’ gut microbiota composition was dominated by the class Bacteroidia (20%), Oxyphotobacteria (19%), and Alphaproteobacteria (15%), while in pregnant females Oxyphotobacteria (27%) was the most abundant class followed by Bacteroidia (16%), and Alphaproteobacteria (11%). Non-reproductive adult females, adult males, and young males’ gut bacteria were dominated by Gammaproteobacteria (51%, 60% and 46% respectively) and Bacilli (30%, 26% and 41% respectively) (Fig 5). The genus *Escherichia-Shigella* was the most abundant among juveniles and adults, followed by *Lactococcus* (Figs 6 and 7)

The PERMANOVA analysis with 1000 permutations and the weighted unifrac distance showed no significant difference between the fecal microbiota composition of pregnant females and lactating females or between juveniles and adults; the strongest differences were between pregnant and lactating females and adult males and females followed by pregnant and lactating females and juveniles (Table 2) (Fig 2). The PCoA showed that the bacterial community structure in pregnant and lactating females is different from that found in non-reproductive adult females, adult males and juvenile males. The PCoA grouped the fecal microbiota compositions of pregnant and lactating females together, differentiating them from the rest of the population (non-reproductive adult males and females and juvenile males) (Fig 3).

**Table 2:** PERMANOVA analysis with 1000 permutations and the weighted unifrac distance. In the pairwise mode, the p-value was adjusted using the false discovery rate (FDR) method. The significant comparisons at a p.value 0.05 are marked with asterisk. The permutest analysis was not significant (p.value = 0.149), thus the PERMANOVA result is reliable because the dispersion of the groups is homogeneous.

**Pairwise**	**Model *F***	***R***^**2**^	**FDR-adjusted *P* value**
Adult vs Lactating	4.8059527	0.17928677	0.006*
Adult vs Pregnant	4.3205027	0.1641497	0.006*
Adult vs Young	0.5715419	0.02778313	1
Lactating vs Pregnant	0.6055095	0.05709386	1
Lactating vs Juvenile	2.6686511	0.2501395	0.024*
Pregnant vs Juvenile	2.4378676	0.23355992	0.042*
**Single**	**Model *F***	**R**^**2**^	**p.value**
Stage	2.8305	0.22061	0.001*

Bacteroidetes, Cyanobacteria, Alphaproteobacteria, Gammaproteobacteria, and Firmicutes had high abundances in pregnant females. In juveniles, Firmicutes was the most abundant, followed by Gammaproteobacteria. In adults of both sexes Gammaproteobacteria were more abundant than Firmicutes (Figs 4 and 5).

**Fig 8:**
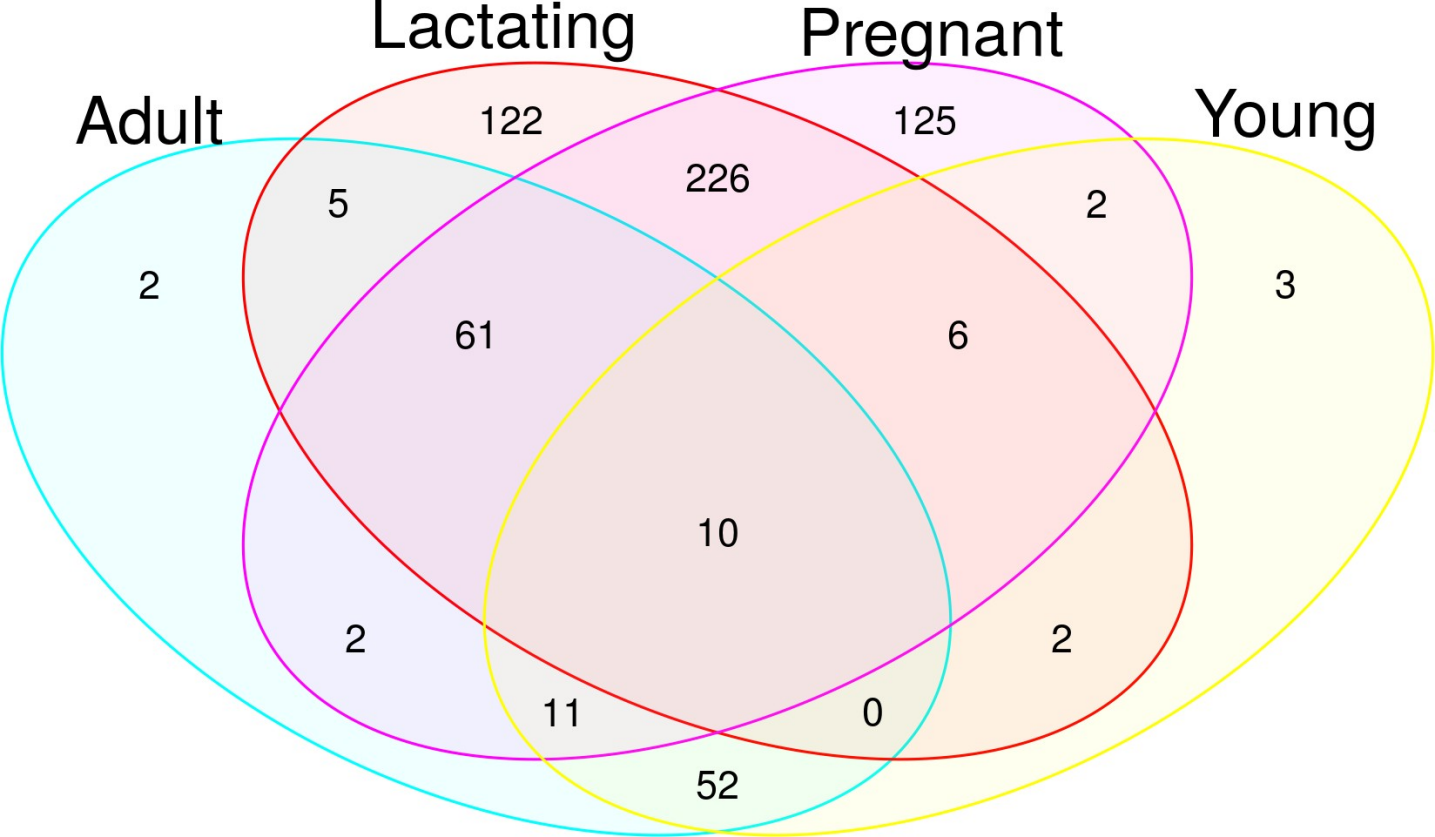
Venn diagram of different reproductive stages of *L*. *yerbabuenae*.

The microbiota of the non-reproductive adult population was dominated by Gammaproteobacteria and Firmicutes. Reproductive females had a much more diverse microbiota, with a clear increase in phyla such as Cyanobacteria, Bacteroidetes and Alphaproteobacteria. Esherichia-Shigella bacteria were one of the main contributors to the microbiota of non-reproductive adults (Fig 6). The pregnant and lactating females shared 54 ASV at the Family level, juveniles and adults (of both sexes) shared 5 (Figs 7 and 8), and no families were shared by all four reproductive stages.

A Venn diagram (Fig 8) of microbiota composition shows more clearly how pregnant and lactating females differed from the rest of the population, with only 10% of their microbiota composition shared with the other stages. Interestingly, juveniles had 79% similarity in microbial composition to non-reproductive adults. Pregnant and lactating females shared 226 ASV; there were 125 representative ASV for pregnancy and 122 representative ASV of lactation. Juveniles and adults shared 52 ASV, and there were 2 ASV representative of adults and 3 representative of juveniles (Fig 8).

## Discussion

### Pregnant and lactating females

The analysis of the fecal microbiota of *Leptonycteris yerbabuenae* individuals in different reproductive stages shows a higher microbial diversity in pregnant and lactating females compared to non-reproductive females, males, and juvenile bats. This could be associated with their increased energy, protein and calcium requirements [18]. Pregnancy (in small mammals) results in the rearrangement of several organs including the stomach and the growth of the intestine [18]. This leads to an increased surface area for colonization by bacteria, which has been reported as an increase in the bacterial carrying capacity during pregnancy in humans and mice [15] and could explain the increase in microbial diversity among pregnant and lactating females. In *L*. *yerbabuenae*, there is a 2.4-fold increase in the diversity of plants consumed by pregnant and lactating females compared to non-reproductive females and males [16]. When there is little nectar available, reproductive females can consume fruits and insects, and females (but not males) of this species [17]. The diet of *L*. *yerbabuenae* has been widely studied, and while it is considered a strictly nectarivorous species [59], the microbiota data reported here suggest a dietary shift during pregnancy and lactation [15]. Bats are adapted to seasonal changes, and can adapt to food resources depending on the food available in the environment [59].

These changes in the gastrointestinal tract in reproductive female bats could suggest that an increase in the diversity of foods consumed and the assimilation of new food sources are the main physiological and morphological responses to the higher energetic demands during pregnancy and lactation [60]. Furthermore, the energy requirements of migration to the pregnancy and lactation caves to which females are phylopatric, imposes additional stress. These high energy requirements may be offset by shifting from a nectar-specialist diet to a more generalist one. During pregnancy, levels of progesterone and estrogen increase, influencing the growth of some bacteria, and some of the changes during pregnancy are similar to changes that occur with illnesses such as obesity and diabetes, which can lead to changes in the microbiota [15].

*L*. *yerbabuenae*’s physiological restrictions on hydrolyzing sucrose imposes physiological limitations due to the lack or inefficiency of sacarase to use this sugar as an energy source [61]. Our results show an increased abundance of Bacteroidetes in pregnant and lactating females. This change may be due to the increased requirements of the host to hydrolyze polysaccharides [62], since intestinal Bacteroidetes are specialized in the degradation of plant-derived polymers, such as plant cell wall compounds (e.g., cellulose, pectin, and xylan) [63].

There is a clear increase in the abundance of Bacteroidetes in both pregnant and lactating females compared to the rest of the population, this change in composition may be considered a host-microbiota adaptation determined by physiological changes during these reproductive stages that result in significant benefits to the host, increasing their nutrient adsorption capacity and energy intake. The phylum Bacteroidetes is a very diverse bacterial phylum, but generally the interactions between Bacteroidetes and their animal hosts are considered mutualistic rather than commensal, since both the bacterium and host receive fitness benefits from the association [63]. The bacteria-mediated fermentation of these foods and host-derived polysaccharides in the colon lead to the release of volatile short-chain fatty acids (mainly acetate, propionate, and butyrate). The assimilation of short-chain fatty acids (SCFA) produced by microbial fermentation of polysaccharides can contribute more than 50% of the total caloric supply [64]. Hence, the presence of Bacteroidetes optimizes the extraction of energy from the diet, likely helping to provide an additional source of energy to pregnant and lactating females. Some members of this phylum can have strong pathogenic behavior, but the emergence of an infection seems to be linked to the assemblage of pathogens in bacterial consortia more than to the individual action of a particular species [65].

The change in the microbiota composition in pregnant and lactating females can be considered a short-term microbiota-host adaptation driven by the physiological changes during these reproductive stages, and which is beneficial because it increases their nutrient adsorption capacity and energy uptake. At the end of this cycle, the organs that were modified during pregnancy return to their non-reproductive state, reducing the size of the gut and the associated bacterial diversity [18]. Cyclical changes in the microbiota have been observed previously, such as seasonal changes marked by food availability [22].

There is no evidence to suggest that the microbiota varies between sexes of non-reproductive adults. Males do not present the changes to the intestine that females do; this suggests that after weaning, when females’ energetic demands are reduced, their foraging behavior reverts to that of a nectar specialist. More studies are needed to test this hypothesis and more thorough studies on food availability and diet would be very informative in this context. On the other hand, while males from this species do spend energy on the development of the dorsal patch, a secondary sexual organ developed during mating season [66], without the excessive energy requirements imposed by giving birth they are likely consistently nectar-feeding specialists throughout their life-history and thus more effective pollinators of agaves and cacti. This hypothesis is consistent with reports by Ibarra-López [67] of Central-population males feeding mainly on two Cactaceae and Agavaceae families. In contrast, a maternity and lactation colony of *L*. *yerbabuenae* in Chiapas, Mexico was found to feed from seven different families and 19 species of plants; it was also reported that the diversity of plant species consumed was lower in lactating females than in pregnant females [16]. These findings are of relevance to conservation management planning of this species, including ecological restoration for mezcal and tequila production, alcoholic beverages of significant economic importance in Mexico.

### Microbiota composition among non-reproductive *L*. *yerbabuenae*

Juvenile bats share 79% of their microbiota with non-reproductive adults and have a less diverse microbiota. In both juvenile and non-reproductive adult males and females, the phylum Firmicutes has a high relative abundance. Eight out of nine genera within this phylum recovered from this microbiota study are SCFA fermenters (genera *Solobacterium*, *Lactococcus*, *Enterococcus*, *Gemella*, *Streptococcus*, *Weissella*, *Anaerococcus* and *Staphylococcus*) [68]. A high proportion of the SCFA produced by microbial fermentation of indigestible carbohydrates in the large intestine is absorbed by the host. Thus, microbial activity contributes energy to the host (estimated to be around 10% of the calories obtained from the diet) that would otherwise be lost through excretion of undegraded substrate in the feces [6]. Increased SCFA concentration may also increase the solubility of certain minerals such as calcium and enhance absorption and expression of calcium-binding proteins. Bacteria of the phylum Firmicutes have a lower ability for polysaccharide degradation [69] and are better known for their production of butyric acid. Butyrate-producing bacteria play an important role in the human colon, supplying energy to the gut epithelium and regulating host cell responses [70]. Butyrate-producing gut bacteria are an important component of the microbiota, in terms of both abundance and functionality [70].

The change in diet and foraging behavior among the different reproductive stages determine the microbiota composition, shaping their physiological requirements. Adults and young could require the production of SCFA to aid calcium absorption [6]. Changes in the intestinal microbial metabolism following the consumption of inulin fructans have also been shown to benefit bone health by increasing calcium absorption, while B-glucans may lower total cholesterol levels [6]. There might be a higher intake of mono-saccharides in non-reproductive females and males due to differences in foraging. Inulin fructans affect gastrointestinal functions not because of their physico-chemical properties, but rather because of their biochemical and physiological attributes. In the colon, they are rapidly fermented to produce SCFA that are good candidates to explain some of the systemic effects of the inulin-type fructans.

### Importance of the microbiota for *L*. *yerbabuenae* reproduction

Bacteria that colonize the mammalian intestine collectively possess a far wider diversity of genes and a larger repertoire of degradative enzymes and metabolic capabilities than their hosts. Fermentation of complex carbohydrates in the intestine involves interactions between community members that include both nutritionally specialized and widely adapted species. Certain dominant species allow them to switch readily between different energy sources in the gut depending on availability, using sophisticated sensing and regulatory mechanisms to control gene expression [6].

The gut microbiota may also influence the expression of host peptides and hormones by producing SCFA via their interactions with free fatty acid receptors, influencing host energy metabolism and appetite regulation [6]. Another potential route linking microbial activity with the host is via the gut-brain axis, a bi-directional communication system based on neural, endocrine and immunological mechanisms [6]. The immune system is influenced by microbial metabolic products, leading to complex interactions between the species composition of the microbiota and the host’s innate and adaptive immune systems that are thought to underlie many probiotic effects [71].

The relevance of the microbiota in *L*. *yerbabuenae* reproduction is not limited to females. In males, the skin microbiota plays an important role in pheromone production during the reproductive season. There is a synchrony between testicle growth and the development of a structure known as the sebaceous patch; a wound-like structure in the interscapular area where fermentative bacteria produce SCFAs, commonly known as pheromones [72]. In light of this, it is worthwhile to further examine the role of the microbiota in reproduction and mating behavior in wildlife.

## Conclusion

This study focused on the diversity of the fecal microbiota in different reproductive stages of the central population of *Leptonycteris yerbabuenae*. Results suggest that the microbiotas of pregnant and lactating females are similar to each other and have higher abundance than juveniles and non-reproductive adults. There was no significant difference in microbiota between juveniles and non-reproductive adults in this population, regardless of the roost in which the adults were captured. Diet is considered to be the main factor shaping the diversity and function of the microbiota [8,9]. The reproductive stage of *L*. *yerbabuenae*, a strictly nectarivorous bat, is important in shaping the microbiota due to physiological changes in the energy requirements during pregnancy and lactation which are consistent with data from the literature that show increased dietary diversity during female reproduction. A relationship exists between the abundances of fecal microbial communities and the different reproductive stages of this nectar-feeding bat. This host-microbiota relationship is more evident in pregnant and lactating females than in other reproductive stages, due to the physiological, anatomical and energy-requirements changes associated with maternity. These requirements trigger a need to consume different foods, and the microbiota is strongly shaped by diet [9,13,51]. Pregnant and lactating females become more generalist feeders to cope with the increased energy requirements, feeding on resources other than nectar. This dietary modification suggests that non-reproductive individuals, which retain a specialist feeding strategy, are more efficient pollinators than reproductive females.

Migration and the segregation between females and males in maternity and bachelor caves might have evolved as a strategy to guarantee resource availability during the high-energy demanding stages of pregnancy and lactation. It may be speculated that the adaptation of gut microbiota could have been important to these evolutionary adaptations of populations. The flexibility of the gut microbiota to shift from a specialist to a generalist diet could have coevolved in reproductive females to increase their fitness and guarantee yearly reproductive success. There are more questions than answers in our understanding of the host-microbiota relationship. Is there a signal that directs the change of abundances in microbiota composition? What is the ecological succession of these communities from one reproductive stage to the other? More research is needed to unravel the patterns of bat-microbiota association, to understand its implications in this species’ ecology, evolution, and life-history.

## Supporting information

S1 Fig Rarefaction curve. (TIF)

S1 Table Alpha diversity indexes per sample. (DOCX)

## References

[1] Ezenwa VO, Gerardo NM, Inouye DW, Medina M, Xavier JB. Animal behavior and the microbiome. Science. 2012; 338(6104):198–199. 10.1126/science.1227412 23066064

[2] Malmuthuge N, Griebel PJ, Guan LL. The gut microbiome and its potential role in the development and function of newborn calf gastrointestinal tract. Front Vet Sci. 2015;2:36 10.3389/fvets.2015.00036 26664965 PMC4672224

[3] Thaiss CA, Zmora N, Levy M, Elinav E. The microbiome and innate immunity. Nature.2016;535(7610):65 10.1038/nature18847 27383981

[4] Nicholson JK, Holmes E, Wilson ID. Gut microorganisms, mammalian metabolism and personalized health care. Nat Rev Microbiol [Internet]. 2005;3(5):431–8. Available from: 10.1038/nrmicro1152 15821725

[5] Van Soest PJ. Nutritional ecology of the ruminant. 2nd ed Ithaca: Cornell University Press; 2004.

[6] Flint HJ, Scott KP, Duncan SH, Louis P, Forano E. Microbial degradation of complex carbohydrates in the gut. Gut Microbes [Internet]. 2012;3(4):289–306. Available from: 10.4161/gmic.19897 22572875 PMC3463488

[7] Sharpton TJ. Role of the Gut Microbiota in Vertebrate Evolution. mSystems [Internet]. 2018;3(2):e00174–17. Available from: 10.1128/mSystems.00174-17 29629413 PMC5881020

[8] Muegge BD, Kuczynski J, Knights D, Clemente JC, Gonzalez A, Fontana L, et al Diet Drives Convergence in Gut Microbiota Functions Across Mammalian Phylogeny and Within Humans. Science (80-) [Internet]. 2011;332(6032):970–4. Available from: 10.1126/science.1198719 21596990 PMC3303602

[9] Fujimura KE, Slusher NA, Cabana MD, Lynch S V. Role of the gut microbiota in defining human health. Expert Rev Anti Infect Ther. 2010;8(4):435–54. 10.1586/eri.10.14 20377338 PMC2881665

[10] Voreades N, Kozil A, Weir TL. Diet and the development of the human intestinal microbiota. Front Microbiol [Internet]. 2014;5 Available from: 10.3389/fmicb.2014.00005 25295033 PMC4170138

[11] Wu GD, Chen J, Hoffmann C, Bittinger K, Chen Y-Y, Keilbaugh SA, et al Linking long-term dietary patterns with gut microbial enterotypes. Science (80-) [Internet]. 2011/09/01. 2011 10 7;334(6052):105–8. Available from: https://www.ncbi.nlm.nih.gov/pubmed/21885731 21885731 10.1126/science.1208344 PMC3368382

[12] Oriach CS, Robertson RC, Stanton C, Cryan JF, Dinan TG. Food for thought: The role of nutrition in the microbiota-gut–brain axis. Clin Nutr Exp [Internet]. 2016;6:25–38. Available from: 10.1016/j.yclnex.2016.01.003

[13] Amato KR. Co-evolution in context: The importance of studying gut microbiotas in wild animals. Microbiota Sci Med. 2013;1(1):10–29.

[14] Brucker RM, Bordenstein SR. The roles of host evolutionary relationships (genus: Nasonia) and development in structuring microbial communities. Evolution (N Y) [Internet]. 2012 Feb 1;66(2):349–62. Available from: 10.1111/j.1558-5646.2011.01454.x 22276533

[15] Koren O, Goodrich JK, Cullender TC, Spor A, Laitinen K, Bäckhed HK, et al Host remodeling of the gut microbiota and metabolic changes during pregnancy. Cell. 2012;150:470–80. 10.1016/j.cell.2012.07.008 22863002 PMC3505857

[16] Riechers Pérez A, Martínez Coronel M, Vidal López R. Consumo de polen de una colonia de maternidad de Leptonycteris curasoae yerbabuenae en Tuxtla Gutiérrez, Chiapas, México. An del Inst Biol Ser Zool. 2003;74(1):43–66.

[17] Sperr E, Caballero-Martínez L, Medellin R, Tschapka M. Seasonal changes in species composition, resource use and reproductive patterns within a guild of nectar-feeding bats in a west Mexican dry forest. J Trop Ecol. 2011;27(2):133–45.

[18] Speakman JR. The physiological costs of reproduction in small mammals. Philos Trans R Soc B Biol Sci [Internet]. 2008;363(1490):375–98. Available from: 10.1098/rstb.2007.2145 PMC2606756 17686735

[19] Edwards SM, Cunningham SA, Dunlop AL, Corwin EJ. The Maternal Gut Microbiome During Pregnancy. MCN Am J Matern Child Nurs. 2017;42(6):310–317. 10.1097/NMC.0000000000000372 28787280 PMC5648614

[20] Gohir W, Whelan FJ, Surette MG, Moore C, Schertzer JD, Sloboda DM. Pregnancy-related changes in the maternal gut microbiota are dependent upon the mother's periconceptional diet. Gut Microbes. 2015;6(5):310–320. 10.1080/19490976.2015.1086056 26322500 PMC4826136

[21] Roager HM, Licht TR, Poulsen SK, Larsen TM, Bahl MI. Microbial enterotypes, inferred by thePrevotella-to-Bacteroides ratio, remained stable during a 6-month randomized controlled diet intervention with the new Nordic diet. Appl Environ Microbiol.2014;80:1142–1149. 10.1128/AEM.03549-13 24296500 PMC3911217

[22] Smits SA, Leach J, Sonnenburg ED, Gonzalez CG, Lichtman JS, Reid G, et al Seasonal cycling in the gut microbiota of the Hadza hunter-gatherers of Tanzania. Science (80-). 2017;357(6353):802–806. 28839072 10.1126/science.aan4834 PMC5891123

[23] Clemente JC, Pehrsson EC, Blaser MJ, Sandhu K, Gao Z, Wang B, et al The microbiota of uncontacted Amerindians. Sci Adv [Internet]. 2015 4 1;1(3):e1500183 Available from: http://advances.sciencemag.org/content/1/3/e1500183.abstract 10.1126/sciadv.1500183 PMC4517851 26229982

[24] Obregon-Tito AJ, Tito RY, Metcalf J, Sankaranarayanan K, Clemente JC, Ursell LK, et al Subsistence strategies in traditional societies distinguish gut microbiotas. Nat Commun [Internet]. 2015 3 25;6:6505 Available from: 10.1038/ncomms7505 25807110 PMC4386023

[25] Hernández-Gómez O, Hoverman JT, Williams RN. Cutaneous Microbial Community Variation across Populations of Eastern Hellbenders (Cryptobranchus alleganiensis alleganiensis). Front Microbiol [Internet]. 2017 7 21;8:1379 Available from: 10.3389/fmicb.2017.01379 28785252 PMC5519570

[26] Grice EA, Kong HH, Conlan S, Deming CB, Davis J, Young AC, et al Topographical and temporal diversity of the human skin microbiota. Science (80-) [Internet]. 2009 5 29;324(5931):1190–2. Available from: https://www.ncbi.nlm.nih.gov/pubmed/19478181 19478181 10.1126/science.1171700 PMC2805064

[27] Gao Z, Tseng C, Pei Z, Blaser MJ. Molecular analysis of human forearm superficial skin bacterial biota. Proc Natl Acad Sci [Internet]. 2007 2 20;104(8):2927 LP-2932. Available from: 10.1073/pnas.0607077104 17293459 PMC1815283

[28] Grieneisen LE, Livermore J, Alberts S, Tung J, Archie EA. Group Living and Male Dispersal Predict the Core Gut Microbiota in Wild Baboons. Integr Comp Biol. 2017;57:770–785. 10.1093/icb/icx046 29048537 PMC5886331

[29] Valiente-Banuet A, Arizmendi MDC, Rojas-Martínez A, Domínguez-Canseco L. Ecological relationships between columnar cacti and nectar-feeding bats in Mexico. J Trop Ecol [Internet]. 1996;12(01):103–19. Available from: 10.1017/s0266467400009330

[30] McCracken GF, Bradbury JW. Social organization and kinship in the polygynous bat Phyllostomus hastatus. Behav Ecol Sociobiol [Internet]. 1981;8(1):11–34. Available from: 10.1007/BF00302840

[31] Ortega J, Castro-Arellano I. Artibeus jamaicensis. Mamm Species. 2001;3(662):1–9.

[32] Stoner KE, Karla AS, Roxana CF, Quesada M. Population dynamics, reproduction, and diet of the lesser long-nosed bat (Leptonycteris curasoae) in Jalisco, Mexico: implications for conservation. Biodivers Conserv. 2004;12:357–73.

[33] Sánchez R, Medellín RA. Food habits of the threatened batLeptonycteris nivalis(Chiroptera: Phyllostomidae) in a mating roost in Mexico. J Nat Hist [Internet]. 2007;41(25–28):1753–64. Available from: 10.1080/00222930701483398

[34] Fleming TH, Nassar J. Population biology of the lesser long-nosed bat Leptonycteris curasoae in Mexico and northern South America In: Fleming TH, Valiente-Banuet A, editors. Columnar cacti and their mutualists: evolution, ecology, and conservation. Tucson: University of Arizona Press; 2002 p. 283–305.

[35] Hayward BJ, Cockrum EL. The Natural History of the Western Long-nosed Bat: Leptonycteris sanborni. Western New Mexico University; 1971.

[36] Ceballos G, Fleming TH, Chavez C, Nassar J. Population Dynamics of Leptonycteris curasoae (Chiroptera: Phyllostomidae) in Jalisco, Mexico. J Mammal [Internet]. 1997;78(4):1220–30. Available from: 10.2307/1383065

[37] Lendell Cockrum E. Seasonal distribution of northwestern populations of the long-nosed bats, Leptonycteris sanborni family Phyllostomidae. An del Inst Biol Ser Zool [Internet]. 1991 1 14;62(2):181–202. Available from: https://www.redalyc.org/articulo.oa?id=45862206

[38] Humphrey SR. Revisión taxonómica de los murciélagos magueyeros del género Leptonycteris (Chiroptera: Phyllostomidae). Instituto de Ecología; 1988.

[39] Arita HT. Spatial Segregation in Long-Nosed Bats, Leptonycteris nivalis and Leptonycteris curasoae, in Mexico. J Mammal [Internet]. 1991;72(4):706–14. Available from: 10.2307/1381831

[40] Rojas-Martínez A, Valiente-Banuet A, del Coro Arizmendi M, Alcántara-Eguren A, Arita HT. Seasonal distribution of the long-nosed bat (Leptonycteris curasoae) in North America: does a generalized migration pattern really exist? J Biogeogr [Internet]. 1999;26(5):1065–77. Available from: 10.1046/j.1365-2699.1999.00354.x

[41] Morales-Garza MR, Arizmendi M del C, Campos JE, Martínez-Garcia M, Valiente-Banuet A. Evidences on the migratory movements of the nectar-feeding bat Leptonycteris curasoae in Mexico using random amplified polymorphic DNA (RAPD). J Arid Environ [Internet]. 2007;68(2):248–59. Available from: 10.1016/j.jaridenv.2006.05.009

[42] Rojas-Martínez A, Godínez-Alvarez H, Valiente-Banuet A, Arizmendi M del C, Sandoval Acevedo O. Frugivory diet of the lesser long-nosed bat (Leptonycteris yerbabuenae), in the Tehuacán Valley of central Mexico. Therya [Internet]. 2012;3(3):371–80. Available from: 10.12933/therya-12-94

[43] García E. Modificaciones al sistema de clasificación climática de Köppen. Universidad Nacional Autónoma de México. Mexico city, Mexico; 1973.

[44] Hoffman A, Palacios-Vargas JG, Morales-Malacara JB. Manual de Bioespeleología. Universidad Nacional Autónoma de México; 1986 274 p.

[45] Rzedowski J. Vegetación de México. Mexico City: Limusa; 1978.

[46] Boyás DC. Determinación de la productividad, composición y estructura de las comunidades arbóreas del estado de Morelos en base a unidades ecológicas. Universidad Nacional Autónoma de México; 1992.

[47] Fuentes L. Tamaño y composición de dos colonias de maternidad del Murciélagos Myotis Velifer en el Estado de Morelos. Universidad Autónoma del Estado de Morelos; 2011.

[48] Kunz TH, Betke M, Hristov NI, Vonhof MJ. Methods for assessing colony size, population size, and relative abundance of bats In: Kunz TH, Parsons S, editors. Ecological and behavioral methods for the study of bats. 2nd ed Baltimore, Maryland: Johns Hopkins University Press; 2009 p. 133–57.

[49] Kunz TH, Wemmer C, Hayssen V. Sex, age and reproductive condition In: Wilson DE, Cole FR, Nichols JD, Rudran R, Foster MS, editors. Measuring and Monitoring Biological Diversity Standard Methods for Mammals. Washington, DC: Smithsonian Institution Press; 1996 p. 279–290.

[50] Caporaso JG, Lauber CL, Walters WA, Berg-Lyons D, Huntley J, Fierer N, et al Ultra-high-throughput microbial community analysis on the Illumina HiSeq and MiSeq platforms. ISME J [Internet]. 2012;6(8):1621–4. Available from: 10.1038/ismej.2012.8 22402401 PMC3400413

[51] Carrillo-Araujo M, Taş N, Alcántara-Hernández RJ, Gaona O, Schondube JE, Medellí-n RA, et al Phyllostomid bat microbiota composition is associated to host phylogeny and feeding strategies. Front Microbiol [Internet]. 2015;6:447 Available from: 10.3389/fmicb.2015.00447 26042099 PMC4437186

[52] Callahan BJ, McMurdie PJ, Rosen MJ, Han AW, Johnson AJA, Holmes SP. DADA2: High-resolution sample inference from Illumina amplicon data. Nat Methods [Internet]. 2016;13(7):581–3. Available from: 10.1038/nmeth.3869 27214047 PMC4927377

[53] Rognes T, Flouri T, Nichols B, Quince C, Mahé F. VSEARCH: a versatile open source tool for metagenomics. PeerJ [Internet]. 2016;4:e2584 Available from: 10.7717/peerj.2584 27781170 PMC5075697

[54] Katoh K, Standley DM. Mafft multiple sequence alignment software version 7: improvements in performance and usability. Mol Biol Evol. 2013;30(4):772–80. 10.1093/molbev/mst010 23329690 PMC3603318

[55] Price MN, Dehal PS, Arkin AP. Fasttree 2–approximately maximum-likelihood trees for large alignments. PLoS One. 2010;5(3):e9490,. 10.1371/journal.pone.0009490 20224823 PMC2835736

[56] McMurdie PJ, Holmes S. phyloseq: An R Package for Reproducible Interactive Analysis and Graphics of Microbiota Census Data. PLoS One [Internet]. 2013;8(4):e61217 Available from: 10.1371/journal.pone.0061217 23630581 PMC3632530

[57] Oksanen J, Blanchet FG, Friendly M, Kindt R, Legendre P, McGlinn D, et al vegan: Community Ecology Package. 2015.

[58] Segata N, Izard J, Waldron L, Gevers D, Miropolsky L, Garrett WS, et al Metagenomic biomarker discovery and explanation. Genome Biol [Internet]. 2011;12(6):R60 Available from: 10.1186/gb-2011-12-6-r60 21702898 PMC3218848

[59] Fleming TH, Nunez RA, da Silveira L, Sternberg L. Seasonal changes in the diets of migrant nectarivorous bats as revealed by carbon stable isotope analysis. Oecologia. 1993;94:72–75. 10.1007/BF00317304 28313861

[60] Reynolds S, Kunz TH. Changes in body composition during reproduction and postnatal growth in the little brown bat,Myotis lucifugus(Chiroptera: Vespertilionidae). Écoscience [Internet]. 2000;7(1):10–7. Available from: 10.1080/11956860.2000.11682565

[61] Ayala-Berdon J, Schondube JE. A Physiological Perspective on Nectar-Feeding Adaptation in Phyllostomid Bats. Physiol Biochem Zool [Internet]. 2011;84(5):458–66. Available from: 10.1086/661541 21897083

[62] Koenig JE, Spor A, Scalfone N, Fricker AD, Stombaugh J, Knight R, et al Succession of microbial consortia in the developing infant gut microbiota. Proc Natl Acad Sci [Internet]. 2011;108(Supplement_1):4578–85. Available from: 10.1073/pnas.1000081107 PMC3063592 20668239

[63] Bäckhed F. Host-Bacterial Mutualism in the Human Intestine. Science (80-) [Internet]. 2005;307(5717):1915–20. Available from: 10.1126/science.1104816 15790844

[64] Carroll EJ, Hungate RE. The magnitude of the microbial fermentation in the bovine rumen. Appl Microbiol [Internet]. 1954 7;2(4):205–14. Available from: https://www.ncbi.nlm.nih.gov/pubmed/13181402 13181402 10.1128/am.2.4.205-214.1954 PMC1056994

[65] Jenkinson HF, Lamont RJ. Oral microbial communities in sickness and in health. Trends Microbiol [Internet]. 2005;13(12):589–95. Available from: 10.1016/j.tim.2005.09.006 16214341

[66] Muñoz-Romo M, Burgos J, Kunz T. Smearing behaviour of male Leptonycteris curasoae (Chiroptera) and female responses to the odour of dorsal patches. Behaviour [Internet]. 2011;148(4):461–83. Available from: 10.1163/000579511x564287

[67] Ibarra-López MP. Comparación de la dieta de dos comunidades de murciélagos nectarívoros: implicaciones ecológicas. University of Guadalajara, Mexico; 2012.

[68] Vos P, Whitman WB, Parte AC. Bergey's manual of systematic bacteriology, Vol. 3 Dordrecht: Springer; 2009.

[69] Kaoutari A El, Armougom F, Gordon JI, Raoult D, Henrissat B. The abundance and variety of carbohydrate-active enzymes in the human gut microbiota. Nat Rev Microbiol [Internet]. 2013;11(7):497–504. Available from: 10.1038/nrmicro3050 23748339

[70] Louis P, Flint HJ. Diversity, metabolism and microbial ecology of butyrate-producing bacteria from the human large intestine. FEMS Microbiol Lett [Internet]. 2009;294(1):1–8. Available from: 10.1111/j.1574-6968.2009.01514.x 19222573

[71] Jarchum I, Pamer EG. Regulation of innate and adaptive immunity by the commensal microbiota. Curr Opin Immunol [Internet]. 2011;23(3):353–60. Available from: 10.1016/j.coi.2011.03.001 21466955 PMC3109238

[72] Martínez-Coronel M, Hortelano-Moncada Y, Corral V, Cuevas LR. Relationship Between Subcutaneous Fat and Reproductive Activity in Males of Leptonycteris yerbabuenae in Los Laguitos Cave, Chiapas, Mexico. Front Reprod Sci Reprod Biol Physiol Biochem male bats [Internet]. 2017;27(1):36–48. Available from: 10.2174/9781681085548117010006

