# Supplementary figures and images for: Fecal microbiota of different reproductive stages of the central population of the lesser-long nosed bat, Leptonycteris yerbabuenae
Source: PLoS One. 2019 Jul 18;14(7):e0219982. doi: 10.1371/journal.pone.0219982 (PMC6639036; doi:10.1371/journal.pone.0219982)

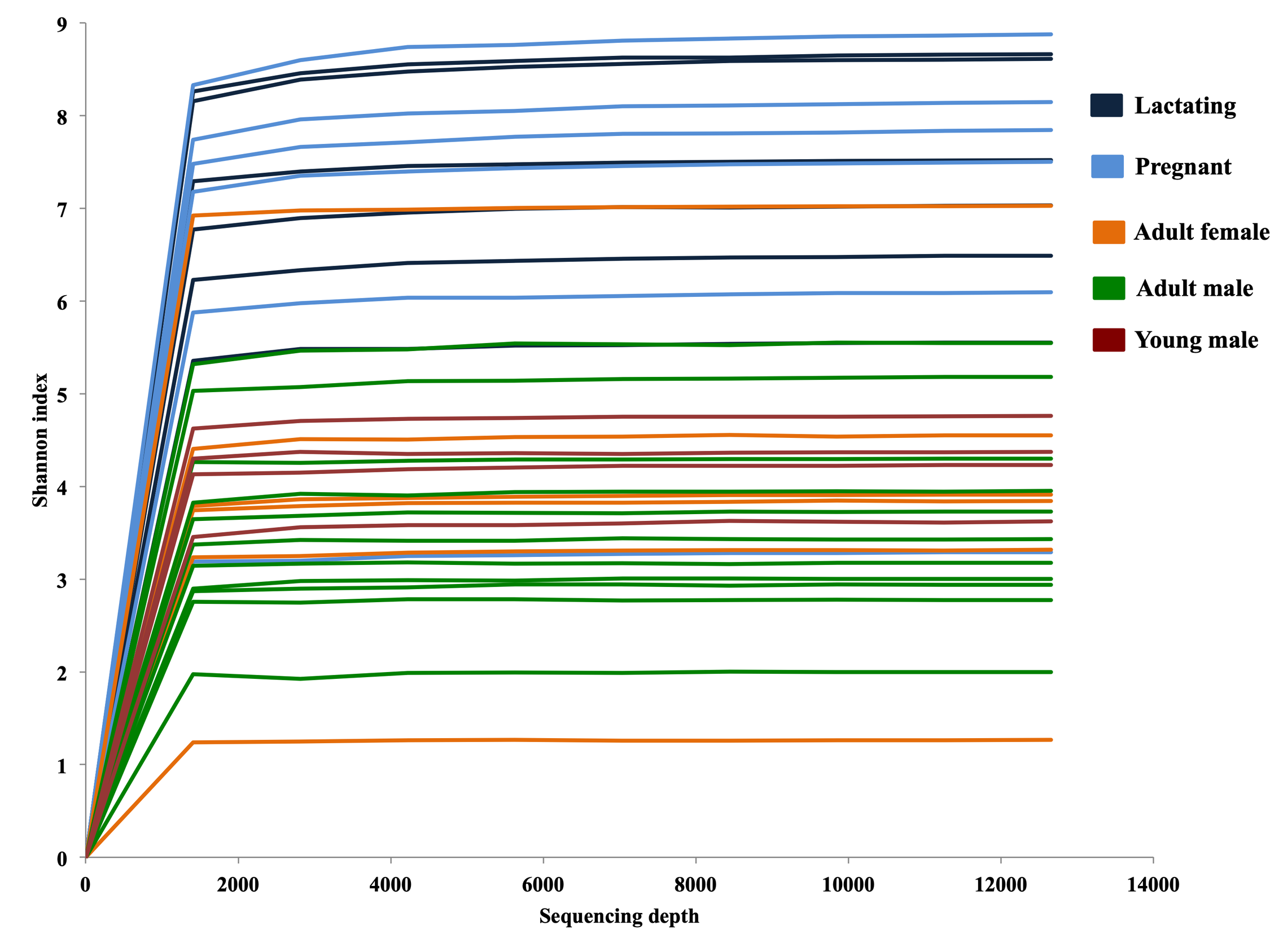

Supplement: S1 Fig — (TIF) [file pone.0219982.s001.tif]
