# Supplementary material for: Fecal microbiota of different reproductive stages of the central population of the lesser-long nosed bat, Leptonycteris yerbabuenae
Source: PLoS One. 2019 Jul 18;14(7):e0219982. doi: 10.1371/journal.pone.0219982 (PMC6639036; doi:10.1371/journal.pone.0219982)
Supplement: S1 Table — (DOCX) [file pone.0219982.s002.docx]

S1 Table. Alpha diversity indexes per sample.

| Sample | Observed | Shannon | Simpson |
| --- | --- | --- | --- |
| LFASM33 | 192 | 2.66277 | 0.833452 |
| LFASM34 | 254 | 2.298562 | 0.615029 |
| LFASM35 | 252 | 2.711633 | 0.789046 |
| LFASM36 | 197 | 4.414347 | 0.975194 |
| LFASM41 | 318 | 3.154164 | 0.863765 |
| LFASM42 | 87 | 0.877845 | 0.314852 |
| LFASM43 | 247 | 4.871824 | 0.985685 |
| LFBSG57 | 654 | 4.497956 | 0.956521 |
| LFBSG69 | 589 | 4.874905 | 0.977651 |
| LFBSG73 | 472 | 3.849837 | 0.883921 |
| LFBSG74 | 870 | 6.004498 | 0.994256 |
| LFBSG77 | 970 | 5.970282 | 0.993683 |
| LFBSG83 | 558 | 5.213897 | 0.988842 |
| LFPSG62 | 508 | 4.225141 | 0.946968 |
| LFPSG65 | 889 | 5.646526 | 0.987316 |
| LFPSG67 | 305 | 2.282396 | 0.611783 |
| LFPSG71 | 897 | 5.436635 | 0.988481 |
| LFPSG79 | 1219 | 6.1544 | 0.993848 |
| LFPSG81 | 734 | 5.199105 | 0.982856 |
| LMASO11 | 393 | 3.591969 | 0.919043 |
| LMASO13 | 196 | 2.038382 | 0.60515 |
| LMASO15 | 36 | 1.924697 | 0.766828 |
| LMASO17 | 158 | 2.979395 | 0.873616 |
| LMASO19 | 131 | 2.379265 | 0.617169 |
| LMASO21 | 453 | 3.846216 | 0.923915 |
| LMASO23 | 189 | 2.738499 | 0.855198 |
| LMASO3 | 184 | 2.082712 | 0.558829 |
| LMASO5 | 148 | 2.583418 | 0.857301 |
| LMASO7 | 74 | 2.201656 | 0.816004 |
| LMASO9 | 110 | 1.384094 | 0.448628 |
| LMYSO26 | 144 | 3.030557 | 0.9057 |
| LMYSO27 | 243 | 3.301995 | 0.891938 |
| LMYSO28 | 275 | 2.510613 | 0.750429 |
| LMYSO29 | 222 | 2.931992 | 0.875893 |
